# VRK2 identifies a subgroup of primary high-grade astrocytomas with a better prognosis

**DOI:** 10.1186/1472-6890-13-23

**Published:** 2013-10-01

**Authors:** Irene Rodríguez-Hernández, Marta Vázquez-Cedeira, Angel Santos-Briz, Juan L García, Isabel F Fernández, Juan A Gómez-Moreta, Javier Martin-Vallejo, Rogelio González-Sarmiento, Pedro A Lazo

**Affiliations:** 1 Instituto de Biología Molecular y Celular del Cáncer, CSIC-Universidad de Salamanca, Campus Miguel de Unamuno, 37007 Salamanca, Spain; 2 Instituto de Investigación Biomédica de Salamanca-IBSAL, Hospital Universitario de Salamanca, Salamanca, Spain; 3 Unidad de Medicina Molecular, Departamento de Medicina, Universidad de Salamanca, Salamanca, Spain; 4 Departamento de Patología, Hospital Universitario de Salamanca, Salamanca, Spain; 5 Departamento de Neurocirugía, Hospital Universitario de Salamanca, Salamanca, Spain; 6 Departamento de Estadística, Universidad de Salamanca, Salamanca, Spain

**Keywords:** Astrocytoma, Glioblastoma, VRK1, VRK2, Immunohistochemistry, Prognosis

## Abstract

**Background:**

Malignant astrocytomas are the most common primary brain tumors and one of the most lethal among human cancers despite optimal treatment. Therefore, the characterization of molecular alterations underlying the aggressive behavior of these tumors and the identification of new markers are thus an important step towards a better patient stratification and management.

**Methods and results:**

VRK1 and VRK2 (Vaccinia-related kinase-1, -2) expression, as well as proliferation markers, were determined in a tissue microarray containing 105 primary astrocytoma biopsies. Kaplan Meier and Cox models were used to find clinical and/or molecular parameters related to overall survival. The effects of VRK protein levels on proliferation were determined in astrocytoma cell lines. High levels of both protein kinases, VRK1 or VRK2, correlated with proliferation markers, p63 or ki67. There was no correlation with p53, reflecting the disruption of the VRK-p53-DRAM autoregulatory loop as a consequence of p53 mutations. High VRK2 protein levels identified a subgroup of astrocytomas that had a significant improvement in survival. The potential effect of VRK2 was studied by analyzing the growth characteristics of astrocytoma cell lines with different EGFR/VRK2 protein ratios.

**Conclusion:**

High levels of VRK2 resulted in a lower growth rate suggesting these cells are more indolent. In high-grade astrocytomas, VRK2 expression constitutes a good prognostic marker for patient survival.

## Background

Malignant astrocytomas represent 70% of adult malignant primary brain tumors and are one of the most devastating cancers [1]. Astrocytomas are classified in four grades on the basis of histological and clinical criteria established [2]. Grade I astrocytomas occur more in children, are curable by surgery and might represent a separate disease from the astrocytomas of other grades [3], however, virtually all high-grade astrocytomas eventually progress and locally relapse regardless of improved diagnosis and multi-modality treatment [4,5]. For most brain tumors the underlying cause is unknown, but most are the result of a multigenic process. Initiation and progression of astrocytomas are related to their genetic and chromosomal alterations. Malignant astrocytomas have well identified molecular characteristics and a basic pattern of common genetic alterations, which include *PTEN*, *TP53, IDH1/2* and p16 mutations, and *EGFR, CDK4* and *MDM2* amplification [1,6].

The human vaccinia-related kinase (VRK) family of serine-threonine kinases is composed of three members, of which only two (VRK1 and VRK2) are catalytically active kinases [7]. Recently, the VRK family has been implicated in different types of neurologic diseases. VRK1 has been associated with pontocerebellar hypoplasia with ataxias and muscular atrophy symptoms [8,9], and VRK2 has been linked to schizophrenia [10-12] and epilepsy [13]. But most of VRK proteins functional roles have been identified in the context of cancer biology [14]. Nuclear VRK1 regulates cell cycle progression by controlling the exit from G0 phase, such as MYC and FOS [15], and phosphorylates several transcription factors such as p53 [16-18] c-Jun [19], ATF2 [20] and CREB [21], as well as histone H3 facilitating chromatin compaction [22]. VRK1 also regulates cellular responses to radiation mediated by p53 or 53BP1 [23]. In addition, VRK1 and p53 form an autoregulatory loop where p53 downregulates VRK1 by the autophagic pathway [24,25]. VRK1 is thus a new important regulator of cell proliferation [14], and high VRK1 protein levels have been associated with the proliferation phenotype in head and neck squamous cell carcinomas [26] and lung cancer [27].

VRK2 has two isoforms, VRK2A and VRK2B. The full-length protein (known as VRK2A or VRK2) is located in the cytosol anchored to endoplasmic reticulum and mitochondrial membranes [28], while the shorter VRK2B is free and present in cytosol and nucleus [28]. VRK2A effects are mediated mostly by its interactions with scaffold proteins that affect cellular signaling. The interaction of VRK2A with JIP1 is able to partially inhibit the stress response to hypoxia [29] or to the interleukin1β [30]. VRK2 also interacts with the KSR1 scaffold protein and downregulates the signal originated in the EGF/Ras/Raf/Mek/Erk pathway [31,32].

In this work we have tested the potential value of VRK1 and VRK2 protein levels as prognostic markers in low and high-grade astrocytomas, based on the observation that these proteins have an effect on proteins that are known to be affected in the pathogenesis of astrocytomas, such as p53, or EGFR signaling, as well as on other regulators of cell cycle progression.

## Methods

### Patients and samples

The study was performed with 105 adult patients diagnosed with primary astrocytoma grades II to IV. The cases included 25 diffuse low-grade astrocytomas (grade II), 20 anaplastic astrocytomas (grade III) and 60 glioblastomas (grade IV) according to WHO criteria [2]. The characteristics of the patients are summarized in Additional file 1: Tables S1 and Additional file 2: Table S2. All samples were obtained with informed consent for publication of patient data and protocols approved by the ethics and scientific committees of the Hospital Universitario de Salamanca and national guidelines for samples of human origin.

### Tissue microarray and immunohistochemistry

Formalin-fixed paraffin-embedded tissue samples were used to prepare a tissue microarray (TMA) made with a Tissue Arrayer device (Beecher Instrument, MD). Three 1-mm-diameter cylinders of each tumor were included to ensure quality, reproducibility and homogenous staining of the slides. Thus, three TMA blocks were constructed, each containing the 105 astrocytoma tumors along with controls of different tissues. Immunohistochemical staining was performed on these sections using p53 (clone DO-7, Novocastra), p63 (clone 4A4, Dako), ki-67 (clone MIB-1, Dako), VRK1 [33] and VRK2 [28] antibodies. Immunohistochemical staining was evaluated semiquantitatively by a blinded pathologist to clinical or molecular information as follows: ki-67 expression was considered positive when >5% of tumor cells showed immunoreactivity; p53, p63, VRK1 and VRK2 expression were scored positive when >50% of tumor cells showed intense staining.

### DNA extraction and mutation analysis of p53 and IDH1/2 genes

DNA from frozen tumor specimens was extracted by standard phenol/chloroform procedure. All coding exons of *p53*, exon 4 of *IDH1* and exon 4 of *IDH2* genes were screened for mutations by PCR amplification and direct sequencing analysis using an ABI Prism 3100 Genetic Analyzer (Applied Biosystems). Primer sequences are listed in Additional file 3: Table S3.

### MGMT promoter methylation status

The MS-MLPA (methylation-specific multiplex ligation-dependent probe amplification) Kit ME011 (MRC-Holland) was used to detect aberrant methylation in *MGMT* gene promoter region using probes that recognized sequences containing a methylation-sensitive restriction site HhaI [34]. All reactions were carried out as described by the kit manufacturer using in each reaction 150 ng of tumor DNA. PCR reaction products were separated by capillary gel electrophoresis (ABI Prism 3100 Genetic Analyzer, Applied Biosystems) and quantified using the Genemapper software (Applied Biosystems). MS-MLPA processing was performed using Coffalyser analysis tool as previously described [34].

### Fluorescence in situ hybridization (FISH)

Dual-probe FISH analysis was performed on TMA sections using locus-specific probes for centromere 7/EGFR gene and centromere 10/PTEN gene (Vysis, Dowerners Grove, IL). FISH studies were carried out following well-established methods as described before [35]. Specimens were considered to have an amplification of *EFGR* when more than 20% of tumor cells exhibited an EGFR/CEP7 ratio >2 or inestimable tight clusters of signals of the locus probe; and *PTEN* deletion was defined as more than 20% of tumor cells containing one or no *PTEN* locus signal and the presence of reference CEP signal.

### Cell culture and immunoblot analysis

The glioblastoma cell lines A172, LN18 and LN229 were obtained from the ATCC and were grown in DMEM with 10% fetal calf serum supplemented with glutamine and antibiotics. Cell lysates and immunoblots were performed as previously reported [26,27,31]. VRK2 was detected with a rabbit polyclonal [28,29]; VRK1 with a rabbit polyclonal [33]; EGFR (sc-3,Santa Cruz Biotechnology); ErbB2 (mAb 44E7, Cell Signaling); cyclin D1(sc-450,Santa Cruz Biotechnology); β-actin (mAb AC-15,Sigma).

### Immunofluorescence and confocal microscopy

The subcellular localization of endogenous VRK1 and VRK2 was determined in the indicated cells lines grown on coverslips and stained with the corresponding antibodies as previously reported [28,31,32]. Fluorescence images were captured with a LEICA TCS SP5 DMI-6000B confocal microscope (Leica) and were analyzed with LEICA LAS AF (Leica) and ImageJ (NIH, http://rsb.info.nih.gov/ij) software.

### Statistical analysis

Associations between molecular and clinicopathological features were analyzed using either the Fisher’s exact test or the chi-square contingency test. Survival models were used to identify correlations between molecular, histopathology and clinical parameters and survival data from the 105 astrocytoma patients. Overall survival was estimated from the date of diagnosis until death or last follow-up, and those patients lost during follow-up were censored. Survival times were estimated by the Kaplan Meier method and compared among patient subsets using the log-rank test. Cox proportional hazards model was used to identify independent prognostic factors in glioma patients. A prognostic index was constructed with those significant variables that predict survival using the Cox regression coefficients derived from Cox model [36]. Patients were ranked according to their risk score and divided into two groups using the median risk score as cut-off. The results were reported following the reporting recommendations for tumor marker prognostic studies (REMARK) criteria [37]. Differences with a p-value smaller than 0.05 were considered as statistically significant, and all tests were two-sided. All statistical analyses were performed using SPSS v.18.0 program (SPSS, Inc.).

## Results

### Expression of VRK1 and VRK2 serine-threonine kinases in human astrocytomas and correlations with tumor grade

**Figure 1:**
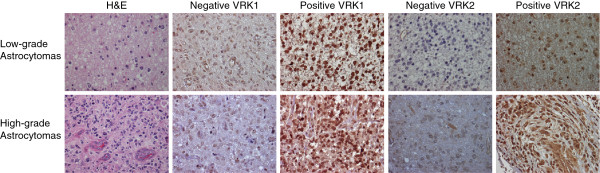
**Illustration of tumor cases representing images of low and high-grade astrocytomas.** The images show hematoxylin and eosin (H&E) staining, as well as negative and positive expression of VRK1 and VRK2 proteins. Images were taken with a magnification of × 400.

The expression of VRK1 and VRK2 proteins was determined in 105 astrocytomas. A representative staining of cases with positive and negative expression of VRK1 and VRK2 proteins, in low and high-grade astrocytoma cases, are shown in Figure 1. VRK1 was mostly detected in the nucleus (Figure 1) [17,33], and there is also a minor VRK1 cytosolic subpopulation located in Golgi (not shown) [18,38]. The main VRK1 nuclear subpopulation was higher in high-grade tumors, including anaplastic astrocytomas and glioblastomas, but did not reach significance (Table 1).

**Table 1:** Expression of markers in low and high-grade astrocytomas, VRK1 expression

	**VRK1 (nuclear staining)**			***P*value**
**Tumor**	**Negative**	**Positive**	**Total**	
**Low-Grade**	15 (62.5%)	9 (37.5%)	24 (100%)	*P* = 0.146
**High-Grade**	36 (45.6%)	43 (54.4%)	79 (100%)	

VRK2 is mostly a cytosolic protein (Figure 1) anchored to the endoplasmic reticulum [28]. VRK2 protein level and tumor grade presented a positive correlation, thus in high-grade astrocytomas its level was significantly higher than in low-grade astrocytomas (Table 2).

**Table 2:** Expression of markers in low and high-grade astrocytomas, VRK2 expression

	**VRK2 (cytoplasmic staining)**			***P*value**
**Tumor**	**Negative**	**Positive**	**Total**	
**Low-Grade**	14 (58.3%)	10 (41.7%)	24 (100%)	*P* = 0.021
**High-Grade**	25 (32.1%)	53 (67.9%)	78 (100%)	

### VRK protein correlations with proliferation markers p63 and ki67

Both VRK1 and VRK2 proteins have been associated with the proliferation phenotype in carcinomas [15,26,27]. Therefore, we also determined the expression of p63 and ki67 proliferation markers as well as p53 expression (Additional file 4: Figure S1), and they were analyzed with respect to VRK1 and VRK2 protein levels. Considering each marker individually, p63 and ki67 significantly correlated with high-grade astrocytomas, reflecting a higher proliferative component in these tumors (Tables 3 and 4), however, the correlation between p63 and ki67 was not significant (data not shown), indicating that these two proliferation markers might vary among tumors. VRK1 expression only correlated with p63 expression in both low and high-grade astrocytomas, while VRK2 expression correlated with ki67 levels only in high-grade astrocytomas (Tables 5 and 6). However, p53 expression was not correlated with tumor grade or with VRK1 or VRK2 expression (data not shown).

**Table 3:** Expression of markers in low and high-grade astrocytomas, p63 expression

	**p63**			***P*value**
**Tumor**	**Negative**	**Positive**	**Total**	
**Low-Grade**	16 (66.7%)	8 (33.3%)	24 (100%)	*P* < 0.001
**High-Grade**	20 (25.3%)	59 (74.7%)	79 (100%)	

**Table 4:** Expression of markers in low and high-grade astrocytomas, Ki-67 expression

	**ki-67**				***P*value**
**Tumor**	**0-5%**	**5-15%**	**>15%**	**Total**	
**Low-Grade**	21 (91.3%)	2 (8.7%)	0 (0.0%)	23 (100%)	*P* < 0.001
**High-Grade**	18 (28.6%)	24 (38.1%)	21 (33.3%)	63 (100%)	

**Table 5:** Correlation of VRK1 with proliferation marker p63

		**p63**			
**Tumor**	**VRK1 (nuclear staining)**	**Negative**	**Positive**	**Total**	***P*value**
**Low-Grade**	**Negative**	13 (54.2%)	2 (8.3%)	24 (100%)	0.007
	**Positive**	3 (12.5%)	6 (25.0%)		
**High-Grade**	**Negative**	15 (19.0%)	21 (26.6%)	79 (100%)	0.002
	**Positive**	5 (6.3%)	38 (48.1%)		

**Table 6:** Correlation of VRK2 with proliferation marker ki-67

		**ki-67**				
**Tumor**	**VRK2 (cytoplasmic staining)**	**0-5%**	**5-15%**	**>15%**	**Total**	***P*value**
**Low-Grade**	**Negative**	13 (56.6%)	1 (4.3%)	0 (0.0%)	23 (100%)	0.742
	**Positive**	8 (34.8%)	1 (4.3%)	0 (0.0%)		
**High-Grade**	**Negative**	9 (14.5%)	2 (3.2%)	5 (8.1%)	62 (100%)	0.005
	**Positive**	8 (12.9%)	22 (35.5%)	16 (25.8%)		

### Mutation profile of astrocytomas

To genetically characterize this astrocytoma series, we determined several alterations in *IDH1/2*, *p53*, *MGMT*, *EGFR* and *PTEN* genes that are known to be frequent in astrocytomas [6].

The frequency of *p53* mutations was significantly higher in low-grade astrocytomas (12/24, 50.0%) than in high-grade astrocytomas (17/73, 23.3%) (p = 0.013). *IDH1/2* mutations were also correlated with low-grade astrocytomas (15/24, 62.5%), and are infrequent in high-grade tumors (3/74, 4.1%) (p < 0.001). On the contrary, loss of *PTEN* locus were more frequent in high-grade astrocytomas (49/65, 75.4%) than in low-grade astrocytomas (4/12, 33.3%) (p = 0.007), and *EGFR* amplification was only detected in high-grade tumors (26/73, 35.6%) (p = 0.002). Finally, *MGMT* promoter methylation status did not show a differential pattern based on tumor grade, 62.5% (15/24) of low-grade astrocytomas and 46.8% (36/77) of high-grade astrocytomas showed *MGMT* hypermethylation. However, we did not find any significant statistical association between these common alterations in astrocytomas and VRK1 or VRK2 protein expression levels (Additional file 5: Table S4).

### High VRK2 protein expression correlated with improved survival and is an independent marker of prognosis

**Figure 2:**
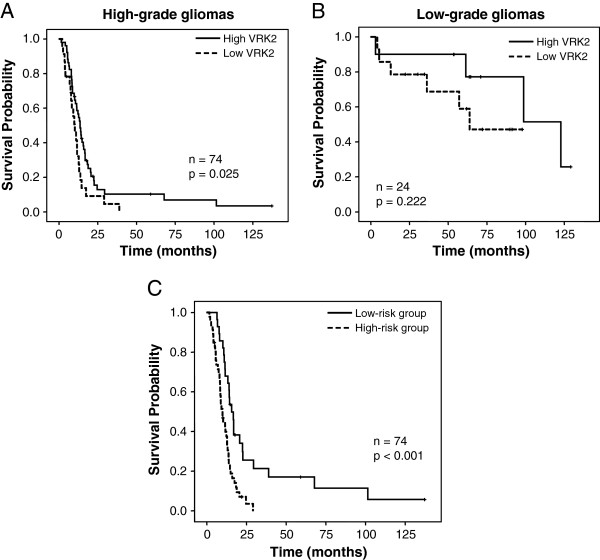
**VRK2 and astrocytoma survival.** Kaplan-Meier estimates of overall survival in **(A)** low-grade astrocytomas and **(B)** high-grade astrocytomas according to VRK2 protein expression. **(C)**. Kaplan-Meier estimates of overall survival for low-risk and high-risk groups, based on the prognostic index combining VRK2 protein level, age and treatment (Table 6) in cases of high-grade astrocytomas.

A survival analysis was performed to investigate the prognostic impact of these proteins in low and high-grade astrocytomas outcome (Figure 2). VRK2 protein expression was significantly associated with survival in high-grade astrocytomas. Those patients with high VRK2 protein expression had a better median overall survival (13.7 months vs. 10.2 months) (Table 7) (Figure 2A). Age (≤60 years vs. >60 years) and treatment (radiotherapy plus chemotherapy vs. radiotherapy alone vs. no treatment) were also significantly associated with improved survival in high-grade astrocytomas by univariate analysis (Table 7). In addition, low-grade astrocytomas with high VRK2 protein levels also had better prognosis but this association did not reach statistical significance probably due to the lower number of cases in this group (Figure 2B).

**Table 7:** VRK2 as independent marker in univariate and multivariate survival analysis in high-grade astrocytomas

	**Univariate analysis**		**Multivariate analysis**	
**Parameter**	**HR (95% C.I.)**	***P*value**	**HR (95% C.I.)**	***P*value**
**VRK2 expression**	1.80 (1.07 – 3.02)	0.025	2.10 (1.24 – 3.58)	0.006
**Age (<60 vs. ≥ 60)**	1.94 (1.15 – 3.26)	0.011	1.80 (1.06 – 3.05)	0.031
**Treatment (Chemo + RT vs. RT alone vs. no treatment)**	1.96 (1.22 – 3.15)	0.003	2.01 (1.22 – 3.30)	0.006

HR: hazard ratio. RT: Radiotherapy. Chemo: Chemotherapy.

Furthermore, multivariate Cox model revealed that VRK2 expression, age and treatment were statistically significant independent predictors of high-grade astrocytoma survival (Table 7). Next, we constructed a prognostic index based on multivariate analysis for predicting patients’ survival with the following prognostic index formula derived from Cox coefficients: 0.744 × VRK2 expression + 0.585 × age + 0.697 × treatment. Patients were divided into low-risk group and high-risk group according to their risk score (Table 8). Thus, patients in the high-risk group had a poor outcome compared to low-risk group of patients (9.8 months vs. 15.7 months) (Figure 2C) (Table 8).

**Table 8:** VRK2 as prognostic index in astrocytomas

**Risk group**	**No. patients %**	**Hazard ratio (95% C.I.)**	**Median survival (months) (95% C.I.)**	***P*value**	**Survival rate**		
					**1-year**	**2-year**	**3-year**
Low-risk	28 (37.8%)	1	15.7 (12.3 – 19.0)	<0.001	67.9%	25.5%	21.3%
High-risk	46 (62.2%)	2.64 (1.53 – 4.55)	9.8 (7.9 – 11.8)		39.8%	7.0%	0.0%

### High VRK2 expression in astrocytoma cell lines restricts cell growth

**Figure 3:**
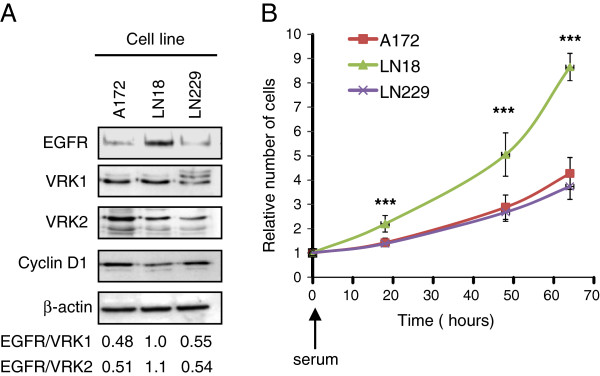
**Protein expression in glioblastoma cell lines.** VRK1, VRK2, EGFR and cyclin D1 protein levels were determined in three glioblastoma cell lines **(A)**. At the bottom the ratio of EGFR expression respect to VRK proteins expression in each cell line is shown. Growth rate of astrocytoma cell lines **(B)**. Cells were seeded in P60 dishes and were serum deprived for 24 hours. At time 0, serum was added and cell number was determined at different time points after serum readdition. To the right is shown the growth curve of each cell line at different time points after serum readdition. The values are the mean of three independent experiments and the standard deviations are shown. *** P < 0.001.

Next, it was determined if glioblastoma cells lines are representative of main tumor and thus can be of use to study glioblastoma signaling characteristics. For this aim the expression and subcellular localization of VRK1 and VRK2 proteins was determined in three glioblastoma cell lines, A172, LN18 and LN229 by immunofluorescence (Additional file 6: Figure S2), and the pattern detected was similar to that observed in astrocytoma biopsies. All cell lines express similar levels of VRK1 and VRK2, but LN18 has a higher level of EGFR and higher EGFR/VRK ratios (Figure 3A), as well as lower cyclin D1. Two cell lines, A172 and LN229, have a higher level of cyclin D1, suggesting that in relative terms there are more cells in G1 phase of the cycle due to their slower progression, a pattern that is inversely correlated with the level of EGFR (Figure 3A). If this interpretation is correct LN18 cells should grow faster than A172 or LN229 cells. Next, it was determined if the growth rate of these three astrocytoma cell lines was in accordance with their EGFR/VRK2 ratio. The LN18 cell line had a higher proliferation rate and a doubling-time of 19 hours (Figure 3B). This cell line has a higher EGFR/VRK2 ratio (Figure 3A) and lower levels cyclin D1 consistent with the progression of cell cycle after serum readdition (Figure 3B). The other cell lines had a doubling-time of 34 hours, and their EGFR/VRK2 ratio was half of that in LN18 cells (Figure 3B).

## Discussion

High-grade astrocytomas overexpress high VRK1 and VRK2 RNA [39] and protein levels (this work). The correlation of VRK1 with p63 expression and the correlation of VRK2 with ki67 levels are very consistent with the association of VRK1 and VRK2 with a proliferation phenotype as it had been reported in head and neck squamous cells carcinomas [15,26]. However, the lack of correlation between VRK1 or VRK2 with p53 levels could be due as a result of mutations in *p53* gene that inactivate the downregulation of VRK1 [18,24,25] that is mediated in the autophagic pathway by DRAM [25].

In our series, low-grade astrocytomas are characterized with *p53* and *IDH1/2* mutations, consistent with known frequencies for this group [6]; whereas *PTEN* loss and *EGFR* amplification that facilitate survival signaling by the resulting constitutive activation of the PI3K pathway [40] were more represented in high-grade astrocytomas [6]. However, none of these alterations were related to VRK1 or VRK2 expression in astrocytomas, suggesting that these proteins could be involved in astrocytoma pathogenesis by different mechanisms.

Furthermore, survival analysis showed that high levels of VRK2 were significantly associated with longer survival in high-grade astrocytomas independently of age or treatment of patients. This association of VRK2 expression to a better prognosis in malignant astrocytomas is likely to be associated to the roles that VRK2 plays in the modulation of mitogenic, stress or apoptosis signaling [29-32]. High levels of VRK2 are known to inhibit the mitogenic signal mediated by activation of the RTK (receptor tyrosine kinase)-Ras-RAF-MEK pathway. VRK2A retains the KSR1 scaffold protein in a membrane-associated form bound to the endoplasmic reticulum and downregulates Erk activation [31,32]. In addition, high VRK2 levels also inhibit the cellular response to stress induced by hypoxia [29] and by inflammatory interleukins [30]. Thus, high VRK2 expression might reduce the strength of the mitogenic signals and contribute to a reduced growth rate as detected in the astrocytoma cell line with higher VRK2 relative level. In that way VRK2 might contribute to the tumor biological characteristics by modulation of neural cells to growth factors. The combined role of these functions might result in a tumor with a lower growth rate and that is also more indolent. This is consistent with the role of high VRK2 level in breast cancer, in which also correlates with tumors having a better prognosis, those that are estrogen and progesterone receptor positive and ERBB2 negative [31,32].

In summary, in this work we have identified the potential value of VRK2 expression as marker of a better prognosis in astrocytomas. The identification of prognostic markers in these lethal tumors is an important issue in order to understand their pathogenesis and might provide basis for the development of new therapeutic strategies.

## Conclusion

High levels of VRK2 protein is an indicator of a better prognosis in primary high-grade astrocytomas.

## Abbreviations

LGA: Low-grade astrocytoma; AA: Anaplastic astrocytoma; GBM: Glioblastomas; VRK: Vaccinia-related kinase

## Competing interests

The authors declare that they have no competing interests.

## Authors’ contributions

IRH and MVC performed the experiments and were involved in data interpretation, data analysis and drafting the manuscript. ASB, JLG and IFF performed the experiments and were involved in data analysis and interpretation. JAGM was involved in sample collection, and acquisition and analysis of clinical data. JMV were involved in data analysis and participated in revising the manuscript. RGS and PAL conceived and designed experiments, participated in analysis and interpretation of data and wrote the manuscript. All authors have read and approved the final manuscript.

## Pre-publication history

The pre-publication history for this paper can be accessed here:

http://www.biomedcentral.com/1472-6890/13/23/prepub

## Supplementary Material

Additional file 1: Table S1 Astrocytoma patient characteristics. Click here for file

Additional file 2: Table S2 Individual characteristics of all astrocytoma cases studied. The information includes tumor localization, patient age and sex, survival and genetic information (p53, IDH1/2 status, EGFR, PTEN and MGMT methylation). Click here for file

Additional file 3: Table S3 Sequence of primers used for detection of mutations in *p53* and *IDH1/2* genes. Click here for file

Additional file 4: Figure S1 Expression level of VRK1, VRK2, p63, Ki-67 and p53 by immunohistochemistry to show cases considered to be either positive or negative. Click here for file

Additional file 5: Table S4 Comparison of VRK1 and VRK2 expression with mutational status in astrocytomas. Click here for file

Additional file 6: Figure S2 Expression of VRK1 and VRK2 in glioblastoma cell lines. Click here for file
