# Supplementary material for: VRK2 identifies a subgroup of primary high-grade astrocytomas with a better prognosis
Source: BMC Clin Pathol. 2013 Oct 1;13:23. doi: 10.1186/1472-6890-13-23 (PMC3849739; doi:10.1186/1472-6890-13-23)
Supplement: Additional file 1: Table S1 — Astrocytoma patient characteristics. [file 1472-6890-13-23-S1.pdf]

Table S1. Astrocytoma patient characteristics

Patients, No. (%)*	Low-Grade Astrocytomas n=25	Anaplastic Astrocytomas n=20	Glioblastomas n=60
**Sex**			
Male	15 (60.0)	13 (65.0)	37 (61.7)
Female	10 (40.0)	7 (35.0)	23 (38.3)
**Median age, years [IQR]**	35 [29.5-47.0]	56 [46.3-66.0]	64 [55.0-69.0]
**Tumor Region**			
Temporal	7 (28.0)	7 (35.0)	23 (38.3)
Frontal	8 (32.0)	6 (30.0)	21 (35.0)
Parietal	3 (12.0)	1 (5.0)	6 (10.0)
Occipital	0 (0.0)	2 (10.0)	5 (8.3)
Other	7 (28.0)	4 (20.0)	5 (8.3)
**Tumor Side**			
Right	9 (36.0)	11 (55.0)	34 (56.7)
Left	9 (36.0)	5 (25.0)	21 (35.0)
Other	7 (28.0)	4 (20.0)	5 (8.3)
**Surgery**			
Total resection	14 (56.0)	14 (70.0)	45 (75.0)
Subtotal resection	9 (36.0)	5 (25.0)	11 (18.3)
Partial resection	2 (8.0)	1 (5.0)	4 (6.7)
**Treatment**			
No treatment	12 (48.0)	4 (20.0)	6 (10.0)
Radiotheraphy	8 (32.0)	8 (40.0)	42 (70.0)
Radiotheraphy and Chemotherapy	5 (20.0)	8 (40.0)	12 (20.0)
**Median Overall Survival, months [IQR]**	61.8 [32.5, 90.6]	13.0 [8.5, 17.4]	11.0 [7.6, 16.9]

IQR: Interquartile range
