# Supplementary material for: VRK2 identifies a subgroup of primary high-grade astrocytomas with a better prognosis
Source: BMC Clin Pathol. 2013 Oct 1;13:23. doi: 10.1186/1472-6890-13-23 (PMC3849739; doi:10.1186/1472-6890-13-23)
Supplement: Additional file 2: Table S2 — Individual characteristics of all astrocytoma cases studied. The information includes tumor localization, patient age and sex, survival and genetic information (p53, IDH1/2 status, EGFR, PTEN and MGMT methylation). [file 1472-6890-13-23-S2.pdf]

TABLE S2. Individual characteristics of all astrocytoma cases. Tumor localization, patient age and sex, genetic information and survival are included.

Case	Histology	Tumor location	Gender	Age	p53 mutation	PTEN Loss	EGFR Gain	IDH1/IDH2 mutation	MGMT Hypermethylation	Patient status	Overall Survival (months)
**1**	Low-grade Astrocytoma	Right parietal lobe	Male	39	No mutation	NA	No	No mutation	No	Dead	63,9
**2**	Low-grade Astrocytoma	Right parietal lobe	Male	32	p.His193Arg (c.578A>G)	Yes	No	IDH1 p.Arg132His (c.395G>A)	No	Alive	22,3
**3**	Low-grade Astrocytoma	Left parietal lobe	Male	29	p.Arg273Cys (c.817C>T)	Yes	No	IDH1 p.Arg132His (c.395G>A)	No	Alive	113,7
**4**	Low-grade Astrocytoma	Right frontal lobe	Male	47	No mutation	NA	No	IDH1 p.Arg132His (c.395G>A)	Yes	Alive	97,9
**5**	Low-grade Astrocytoma	Ventricular	Female	58	No mutation	NA	No	No mutation	No	Dead	12,8
**6**	Low-grade Astrocytoma	Left temporal lobe	Female	25	p.Arg273Cys (c.817C>T)	NA	No	IDH2 p.Arg172Met (c.515G>T)	No	Dead	98,9
**7**	Low-grade Astrocytoma	Right temporal lobe	Female	33	p.Tyr236Cys (c.707A>G)	No	No	IDH1 p.Arg132His (c.395G>A)	No	Dead	57,1
**8**	Low-grade Astrocytoma	Right frontal lobe	Male	31	p.Ile195Thr (c.584T>C)	NA	NA	IDH1 p.Arg132His (c.395G>A)	No	Alive	91,3
**9**	Low-grade Astrocytoma	Left frontal lobe	Female	30	p.Tyr205Cys (c.614A>G)	No	No	IDH1 p.Arg132Cys (c.394C>T)	Yes	Dead	36,2
**10**	Low-grade Astrocytoma	Other hemispheric location	Female	55	p.Arg175His (c.524G>A)	NA	No	IDH1 p.Arg132His (c.395G>A)	Yes	Dead	61,4
**11**	Low-grade Astrocytoma	Right frontal lobe	Male	43	No mutation	NA	NA	IDH1 p.Arg132His (c.395G>A)	Yes	Alive	71,0
**12**	Low-grade Astrocytoma	Right temporal lobe	Female	35	p.Arg273Cys (c.817C>T)	Yes	No	IDH1 p.Arg132His (c.395G>A)	No	Dead	122,9
**13**	Low-grade Astrocytoma	Left frontal lobe	Male	47	No mutation	No	No	IDH1 p.Arg132His (c.395G>A)	Yes	Alive	129,2
**14**	Low-grade Astrocytoma	Left temporal lobe	Female	35	No mutation	NA	No	No mutation	Yes	Alive	72,2
**15**	Low-grade Astrocytoma	Left temporal lobe	Female	35	NA	No	No	NA	NA	Dead	3,0
**16**	Low-grade Astrocytoma	Other hemispheric location	Male	33	No mutation	Yes	No	No mutation	Yes	Dead	5,3
**17**	Low-grade Astrocytoma	Left temporal lobe	Male	52	p.Arg280Gly (c.838A>G)	NA	No	IDH1 p.Arg132His (c.395G>A)	Yes	Alive	34,7
**18**	Low-grade Astrocytoma	Right frontal lobe	Male	62	No mutation	NA	No	No mutation	Yes	Dead	4,1
**19**	Low-grade Astrocytoma	Right frontal lobe	Female	41	p.Arg273Cys (c.817C>T)	NA	NA	IDH1 p.Arg132His (c.395G>A)	Yes	Alive	30,4
**20**	Low-grade Astrocytoma	Left temporal lobe	Male	28	p.Arg175His (c.524G>A); p.Ile195Phe (c.583A>T)	NA	NA	IDH1 p.Arg132His (c.395G>A)	Yes	Alive	64,6
**21**	Low-grade Astrocytoma	Other hemispheric location	Male	40	p.Arg273Cys (c.817C>T)	NA	No	IDH1 p.Arg132His (c.395G>A)	Yes	Alive	63,7
**22**	Low-grade Astrocytoma	Cerebellar hemisphere	Male	15	No mutation	No	NA	No mutation	Yes	Alive	90,0
**23**	Low-grade Astrocytoma	Cerebellar vermis	Female	17	No mutation	No	No	No mutation	No	Alive	61,8
**24**	Low-grade Astrocytoma	Left frontal lobe	Male	29	No mutation	No	No	No mutation	Yes	Alive	53,8
**25**	Low-grade Astrocytoma	Other hemispheric location	Male	49	No mutation	No	No	No mutation	Yes	Alive	53,5
**26**	Anaplastic Astrocytoma	Left temporal lobe	Male	63	No mutation	Yes	No	No mutation	Yes	Dead	4,1
**27**	Anaplastic Astrocytoma	Left occipital lobe	Male	73	No mutation		No	No mutation	Yes	Dead	12,7
**28**	Anaplastic Astrocytoma	Right parietal lobe	Male	74	No mutation	Yes	No	No mutation	Yes	Dead	5,2
**29**	Anaplastic Astrocytoma	Right temporal lobe	Male	48	No mutation	Yes	No	No mutation	No	Dead	15,7
**30**	Anaplastic Astrocytoma	Left frontal lobe	Male	57	No mutation	Yes	Yes	No mutation	No	Dead	14,0
**31**	Anaplastic Astrocytoma	Right temporal lobe	Male	55	No mutation	Yes	Yes	No mutation	No	Alive	17,8
**32**	Anaplastic Astrocytoma	Right occipital lobe	Female	47	No mutation	No	Yes	No mutation	Yes	Dead	10,7
**33**	Anaplastic Astrocytoma	Other hemispheric location	Male	45	No mutation	Yes	Yes	No mutation	No	Dead	8,5
**34**	Anaplastic Astrocytoma	Right frontal lobe	Female	55	No mutation	NA	Yes	IDH1 p.Arg132His (c.395G>A)	Yes	Dead	33,8
**35**	Anaplastic Astrocytoma	Right temporal lobe	Male	68	No mutation	NA	No	No mutation	No	Dead	13,0
**36**	Anaplastic Astrocytoma	Right temporal lobe	Female	66	No mutation	NA	NA	No mutation	No	Dead	13,6
**37**	Anaplastic Astrocytoma	Left frontal lobe	Male	66	p.Arg248Trp (c.742C>T)	No	No	No mutation	Yes	Dead	17,4
**38**	Anaplastic Astrocytoma	Right frontal lobe	Male	43	No mutation	Yes	Yes	No mutation	No	Dead	11,6
**39**	Anaplastic Astrocytoma	Left frontal lobe	Female	37	pVal197Gly (c.590T>G)	NA	No	IDH1 p.Arg132His (c.395G>A)	No	Dead	38,9
**40**	Anaplastic Astrocytoma	Cerebellar hemisphere	Male	46	No mutation	No	No	No mutation	No	Dead	67,9
**41**	Anaplastic Astrocytoma	Right frontal lobe	Female	55	No mutation	NA	Yes	No mutation	No	Dead	11,4
**42**	Anaplastic Astrocytoma	Other hemispheric location	Female	66	No mutation	Yes	No	No mutation	No	Dead	0,5
**43**	Anaplastic Astrocytoma	Right temporal lobe	Male	73	No mutation	Yes	No	No mutation	No	Dead	3,8
**44**	Anaplastic Astrocytoma	Cerebellar hemisphere	Male	14	p.Arg342Stop (c.1024C>T)	No	No	No mutation	No	Dead	14,4
**45**	Anaplastic Astrocytoma	Right temporal lobe	Female	62	No mutation	Yes	No	No mutation	No	Dead	5,0
**46**	*Glioblastoma*	Right frontal lobe	Male	55	No mutation	NA	No	No mutation	No	Dead	2,1

	Multiforme										
**47**	Glioblastoma Multiforme	Left frontal lobe	Male	54	No mutation	No	No	IDH1 p.Arg132His (c.395G>A)	Yes	Alive	137,3
**48**	Glioblastoma Multiforme	Right frontal lobe	Male	76	No mutation	Yes	No	No mutation	Yes	Dead	8,2
**49**	Glioblastoma Multiforme	Right frontal lobe	Male	69	No mutation	NA	No	No mutation	NA	Dead	6,9
**50**	Glioblastoma Multiforme	Left temporal lobe	Male	45	No mutation	No	No	No mutation	Yes	Dead	16,7
**51**	Glioblastoma Multiforme	Right frontal lobe	Female	60	No mutation	Yes	Yes	No mutation	Yes	Alive	14,7
**52**	Glioblastoma Multiforme	Right frontal lobe	Male	64	No mutation	NA	NA	No mutation	Yes	Dead	11,5
**53**	Glioblastoma Multiforme	Left frontal lobe	Male	71	NA	Yes	No	NA	Yes	Alive	21,9
**54**	Glioblastoma Multiforme	Right temporal lobe	Male	70	p.Arg273His (c.818G>A)	NA	NA	No mutation	Yes	Dead	2,7
**55**	Glioblastoma Multiforme	Right occipital lobe	Male	72	No mutation	NA	No	No mutation	No	Dead	8,4
**56**	Glioblastoma Multiforme	Right frontal lobe	Female	79	p.Thr256Ile (c.767C>T)	NA	NA	No mutation	No	Dead	1,6
**57**	Glioblastoma Multiforme	Left parietal lobe	Male	64	No mutation	NA	Yes	No mutation	No	Dead	22,5
**58**	Glioblastoma Multiforme	Right occipital lobe	Female	71	No mutation	Yes	Yes	No mutation	Yes	Alive	10,1
**59**	Glioblastoma Multiforme	Left frontal lobe	Male	51	No mutation	Yes	Yes	No mutation	No	Dead	16,9
**60**	Glioblastoma Multiforme	Left frontal lobe	Female	59	No mutation	No	Yes	No mutation	Yes	Dead	29,5
**61**	Glioblastoma Multiforme	Other hemispheric location	Female	49	No mutation	NA	NA	No mutation	No	Dead	9,8
**62**	Glioblastoma Multiforme	Other hemispheric location	Male	34	p.Arg196Stop (c.586C>T)	No	No	No mutation	Yes	Dead	0,7
**63**	Glioblastoma Multiforme	Right frontal lobe	Male	66	c.673-1G>A	No	No	No mutation	Yes	Dead	24,6
**64**	Glioblastoma Multiforme	Right parietal lobe	Male	56	NA	Yes	Yes	NA	NA	Dead	14,2
**65**	Glioblastoma Multiforme	Right parietal lobe	Male	55	p.Leu257Val (c.769C>G)	NA	No	No mutation	Yes	Dead	8,0
**66**	Glioblastoma Multiforme	Left parietal lobe	Male	52	No mutation	Yes	Yes	No mutation	No	Dead	16,9
**67**	Glioblastoma Multiforme	Right temporal lobe	Female	51	p.Asn131del (c.391delAAC)	Yes	No	No mutation	No	Dead	6,7
**68**	Glioblastoma Multiforme	Right temporal lobe	Male	55	No mutation	NA	No	No mutation	Yes	Dead	0,3
**69**	Glioblastoma Multiforme	Cerebellar vermis	Female	52	No mutation	Yes	No	No mutation	Yes	Dead	29,1
**70**	Glioblastoma Multiforme	Right occipital lobe	Female	67	No mutation	Yes	NA	No mutation	No	Dead	9,8
**71**	Glioblastoma Multiforme	Left temporal lobe	Male	65	p.Val147Asp (c.440T>A)	Yes	No	No mutation	No	Dead	5,6
**72**	Glioblastoma Multiforme	Left parietal lobe	Male	70	No mutation	Yes	No	No mutation	Yes	Dead	8,0
**73**	Glioblastoma Multiforme	Right temporal lobe	Male	76	No mutation	No	Yes	No mutation	Yes	Dead	5,6
**74**	Glioblastoma Multiforme	Right temporal lobe	Male	67	NA	Yes	No	NA	No	Dead	4,1
**75**	Glioblastoma Multiforme	Right occipital lobe	Female	73	No mutation	NA	No	No mutation	Yes	Dead	8,7
**76**	Glioblastoma Multiforme	Right temporal lobe	Female	70	No mutation	Yes	No	No mutation	No	Dead	18,8
**77**	Glioblastoma Multiforme	Left temporal lobe	Male	43	No mutation	No	Yes	No mutation	Yes	Dead	8,3
**78**	Glioblastoma Multiforme	Left temporal lobe	Female	67	p.Ser241Phe (c.722C>T)	Yes	No	No mutation	No	Dead	5,9
**79**	Glioblastoma Multiforme	Right temporal lobe	Male	62	No mutation	No	No	No mutation	No	Dead	0,2
**80**	Glioblastoma Multiforme	Right temporal lobe	Female	66	No mutation	Yes	No	No mutation	No	Dead	20,7
**81**	Glioblastoma Multiforme	Right occipital lobe	Male	69	NA	Yes	Yes	NA	No	Dead	20,4
**82**	Glioblastoma Multiforme	Left temporal lobe	Male	48	No mutation	No	No	No mutation	Yes	Dead	7,4
**83**	Glioblastoma Multiforme	Right frontal lobe	Female	74	NA	Yes	No	NA	NA	Dead	12,3
**84**	Glioblastoma Multiforme	Right temporal lobe	Female	63	p.Ile255Thr (c.764T>C)	No	Yes	No mutation	No	Dead	8,8
**85**	Glioblastoma Multiforme	Right temporal lobe	Male	68	No mutation	No	Yes	No mutation	Yes	Dead	10,2
**86**	Glioblastoma Multiforme	Right frontal lobe	Female	69	No mutation	No	Yes	No mutation	Yes	Dead	14,9
**87**	Glioblastoma Multiforme	Left temporal lobe	Female	35	No mutation	Yes	Yes	No mutation	Yes	Dead	10,1
**88**	Glioblastoma Multiforme	Left temporal lobe	Male	72	p.Met237Ile (c.711G>A)	NA	NA	No mutation	Yes	Dead	11,7
**89**	Glioblastoma Multiforme	Right frontal lobe	Male	62	No mutation	Yes	No	No mutation	Yes	Dead	12,7
**90**	Glioblastoma Multiforme	Left temporal lobe	Male	61	No mutation	Yes	Yes	No mutation	No	Dead	13,8
**91**	Glioblastoma Multiforme	Left temporal lobe	Female	27	No mutation	NA	NA	No mutation	No	Dead	101,5
**92**	Glioblastoma Multiforme	Right frontal lobe	Female	67	NA	NA	NA	NA	No	Dead	14,6
**93**	Glioblastoma Multiforme	Left frontal lobe	Male	54	p.Arg290His (c.869G>A)	Yes	No	No mutation	Yes	Dead	9,2
**94**	Glioblastoma Multiforme	Left frontal lobe	Male	59	No mutation	Yes	Yes	No mutation	No	Dead	6,4
**95**	Glioblastoma Multiforme	Right temporal lobe	Male	69	No mutation	Yes	No	No mutation	No	Dead	16,0
**96**	Glioblastoma Multiforme	Right frontal lobe	Male	72	No mutation	No	No	No mutation	No	Dead	13,3

**97**	Glioblastoma Multiforme	Right parietal lobe	Female	69	No mutation	Yes	No	No mutation	Yes	Dead	3,6
**98**	Glioblastoma Multiforme	Other hemispheric location	Male	65	No mutation	NA	No	No mutation	No	Dead	13,7
**99**	Glioblastoma Multiforme	Other hemispheric location	Female	72	No mutation	Yes	Yes	No mutation	Yes	Dead	18,4
**100**	Glioblastoma Multiforme	Right temporal lobe	Male	61	No mutation	Yes	No	No mutation	No	Alive	4,8
**101**	Glioblastoma Multiforme	Left temporal lobe	Female	64	p.Arg156Gly (c.466C>G)	No	No	No mutation	No	Dead	7,0
**102**	Glioblastoma Multiforme	Left frontal lobe	Female	55	No mutation	Yes	No	No mutation	Yes	Dead	11,0
**103**	Glioblastoma Multiforme	Right temporal lobe	Male	53	No mutation	No	No	No mutation	No	Dead	7,8
**104**	Glioblastoma Multiforme	Left frontal lobe	Female	68	p.Arg282Trp (c.844C>T)	Yes	No	No mutation	Yes	Dead	22,7
**105**	Glioblastoma Multiforme	Right frontal lobe	Male	57	p.Asp259Val (c.776A>T)	NA	No	No mutation	Yes	Alive	59,1
