# Supplementary material for: VRK2 identifies a subgroup of primary high-grade astrocytomas with a better prognosis
Source: BMC Clin Pathol. 2013 Oct 1;13:23. doi: 10.1186/1472-6890-13-23 (PMC3849739; doi:10.1186/1472-6890-13-23)
Supplement: Additional file 3: Table S3 — Sequence of primers used for detection of mutations in p53 and IDH1/2 genes. [file 1472-6890-13-23-S3.pdf]

Table S3.

Sequence of primers used for detection of mutations in *p53* and *IDH1/2* genes.

Primer Name	Primer sequences 5'-3'
**p53 exon 1 F**	GCTCAAGACTGGCGCTAAAA
**p53 exon 1 R**	GTGACTCAGAGAGGACTCAT
**p53 exon 2 F**	GAAGCAGCCATTCTTTTCCT
**p53 exon 2 R**	GGTCCCCAGCCCAACCCTT
**p53 exon 3 F**	GGAGCCGCAGTCAGATCCTA
**p53 exon 3 R**	GGTCCCCAGCCCAACCCTT
**p53 exon 4 F**	CAACGTTCTGGTAAGGACAA
**p53 exon 4 R**	GCCAGGCATTGAAGTCTCAT
**p53 exon 5 F**	GCCGTGTTCCAGTTGCTTTA
**p53 exon 5 R**	AGGAGGGGCCAGACCTAAGA
**p53 exon 6 F**	AGCGCTGCTCAGATAGCGAT
**p53 exon 6 R**	TAAGCAGCAGGAGAAAGCCC
**p53 exon 7 F**	AAGGCGCACTGGCCTCATCTT
**p53 exon 7 R**	GAGGTGGATGGGTAGTAG
**p53 exon 8 F**	GACCTGATTTCCTTACTGCCT
**p53 exon 8 R**	TCTCCTCCACCGCTTCTT
**p53 exon 9 F**	GGAGACCAAGGGTGCAGTAT
**p53 exon 9 R**	GCCCCAATTGCAGGTAAAAC
**p53 exon 10 F**	GGTACTTGAAGTGCAGTTTCT
**p53 exon 10 R**	CAGCTGCCTTTGACCATGAA
**p53 exon 11 F**	CCAGCCTTAGGCCCTTCAAA
**p53 exon 11 R**	TGTCAGTGGGGAACAAGAA
**IDH1 exon 4 F**	CGGTCTTCAGAGAAGCCATT
**IDH1 exon 4 R**	CACATTATTGCCAACATGAC
**IDH2 exon 4 F**	GATGGCGGCTGCAGTGGGACC
**IDH2 exon 4 R**	TAGGCCAGGAGCTCCAGTCG

F: forward, R: reverse.
