# Supplementary material for: VRK2 identifies a subgroup of primary high-grade astrocytomas with a better prognosis
Source: BMC Clin Pathol. 2013 Oct 1;13:23. doi: 10.1186/1472-6890-13-23 (PMC3849739; doi:10.1186/1472-6890-13-23)
Supplement: Additional file 4: Figure S1 — Expression level of VRK1, VRK2, p63, Ki-67 and p53 by immunohistochemistry to show cases considered to be either positive or negative. [file 1472-6890-13-23-S4.pdf]

Figure S1

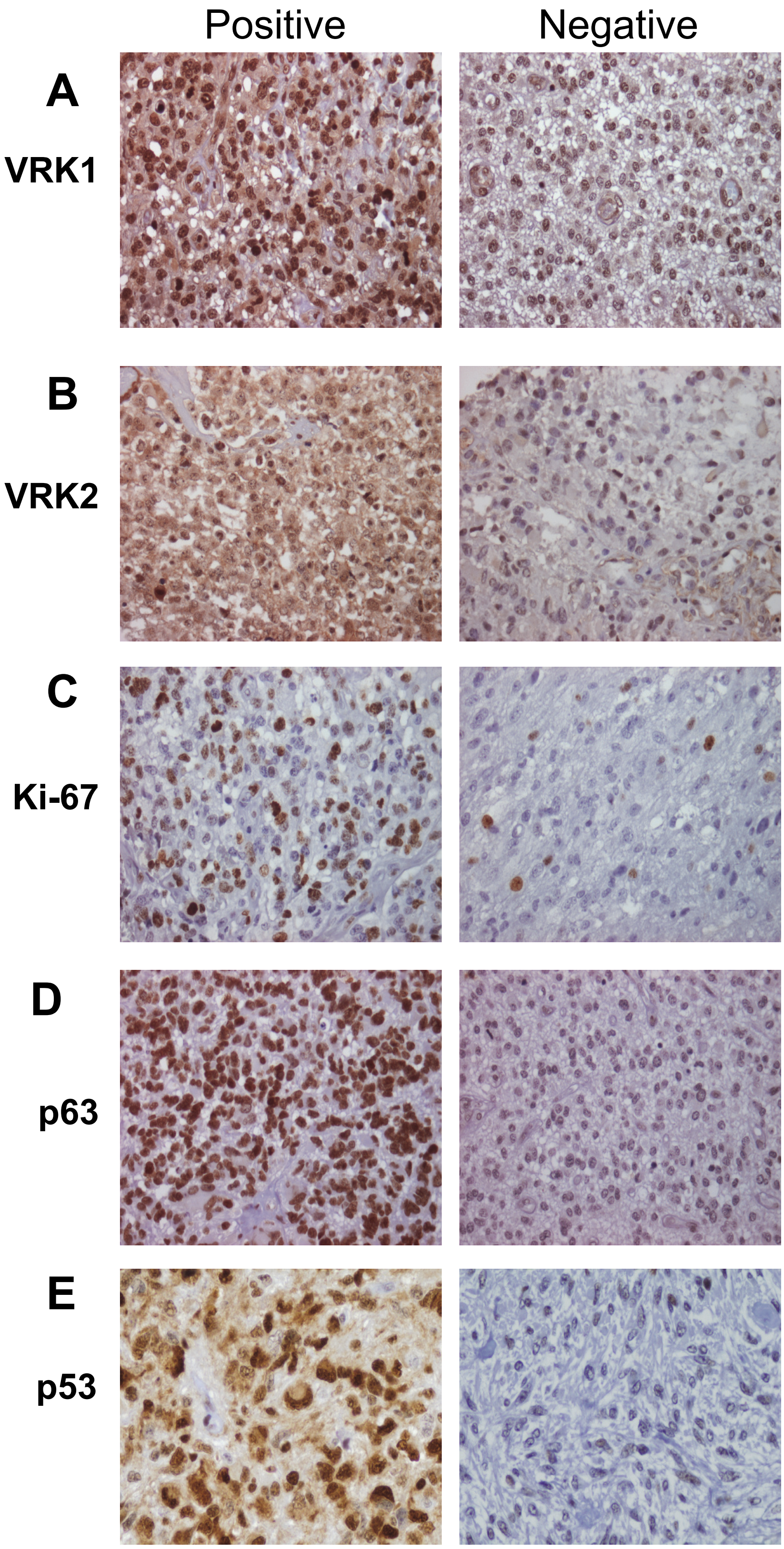

Figure S1. Expression level of different proteins by immunohistochemistry to show cases considered to be either positive (left column) or negative (right column) expressing VRK1, VRK2, p63, Ki-67 and p53 in glioblastomas. Images taken with at x400 magnification..
