# Supplementary material for: VRK2 identifies a subgroup of primary high-grade astrocytomas with a better prognosis
Source: BMC Clin Pathol. 2013 Oct 1;13:23. doi: 10.1186/1472-6890-13-23 (PMC3849739; doi:10.1186/1472-6890-13-23)
Supplement: Additional file 5: Table S4 — Comparison of VRK1 and VRK2 expression with mutational status in astrocytomas. [file 1472-6890-13-23-S5.pdf]

Table S4. Comparison of VRK1 and VRK2 expression with mutational status in astrocytomas.

	VRK1 EXPRESSION			VRK2 EXPRESSION		
	**Negative**	**Positive**	**P-value**	**Negative**	**Positive**	**P-value**
**p53 mutation**			0.443			0.408
**-No mutation**	35 (74.5%)	33 (67.3%)		25 (65.8%)	42 (73.7%)	
**-Mutation**	12 (25.5%)	16 (32.7%)		13 (34.2%)	15 (26.3%)	
**IDH1/2 mutation**			0.717			0.371
**-No mutation**	38 (80.9%)	41 (83.7%)		30 (78.9%)	49 (86.0%)	
**-Mutation**	9 (19.1%)	8 (16.3%)		8 (21.1%)	8 (14.0%)	
**PTEN Deletion**			0.457			0.156
**-No deletion**	8 (26.7%)	16 (34.8%)		11 (40.7%)	12 (25.0%)	
**-Deletion**	22 (73.3%)	30 (65.2%)		16 (59.3%)	36 (75.0%)	
**EGFR Amplification**			0.847			0.122
**-No amplification**	29 (70.7%)	37 (72.5%)		27 (81.8%)	38 (66.7%)	
**-Amplification**	12 (29.3%)	14 (27.5%)		6 (18.2%)	19 (33.3%)	
**MGMT methylation**			0.087			0.504
**-No hypermethylation**	20 (40.8%)	29 (58.0%)		17 (44.7%)	31 (51.7%)	
**-Hypermethylation**	29 (59.2%)	21 (42.0%)		21 (55.3%)	29 (48.3%)	
