# Supplementary material for: VRK2 identifies a subgroup of primary high-grade astrocytomas with a better prognosis
Source: BMC Clin Pathol. 2013 Oct 1;13:23. doi: 10.1186/1472-6890-13-23 (PMC3849739; doi:10.1186/1472-6890-13-23)
Supplement: Additional file 6: Figure S2 — Expression of VRK1 and VRK2 in glioblastoma cell lines. [file 1472-6890-13-23-S6.pdf]

Figure S2

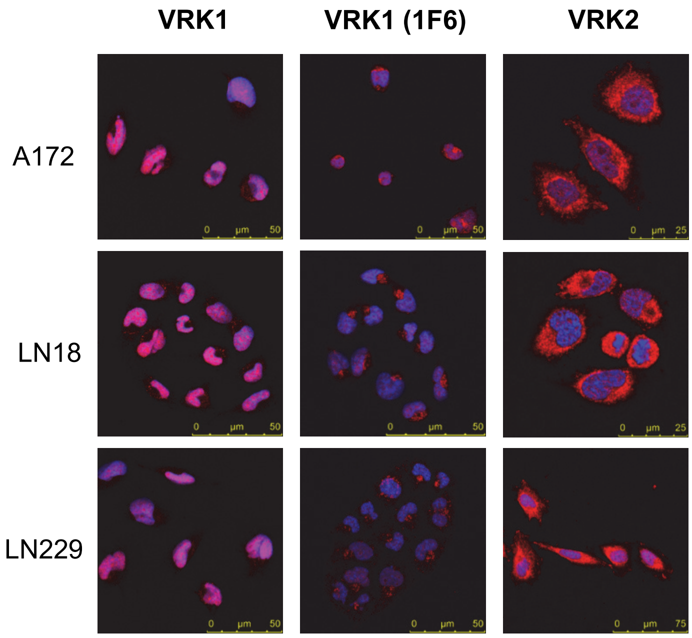

Figure S2. Expression of VRK1 and VRK2 in glioblastoma cell lines. Confocal microscopy showing the subcellular localization of VRK1 determined with rabbit polyclonal (left) or the 1F6 monoclonal (center) and VRK2 (right) detected by immunofluorescence. DAPI was used to identify nuclei. Immunofluorescence methods were as previously reported (Blanco el at 2006). VRK1 is mostly nuclear in the three cell lines (left), although a minor subpopulation could be detected with a different antibody (1F6) in cytosolic Golgi vesicles (central) as expected for normal expression [31]. VRK2 antibody does not discriminate, between the VRK2A and VRK2B isoforms, and in all astrocytoma cell lines VRK2 proteins are cytosolic (right), suggesting they correspond to the VRK2A isoform.
